# Virtual peer teaching in the gross anatomy lab: a format of peer teaching and learning during the COVID-19 pandemic

**DOI:** 10.12688/mep.19178.2

**Published:** 2023-03-09

**Authors:** Abigail C. Hielscher, Stephen Everse

**Affiliations:** 1 Neurological Sciences, University of Vermont, Burlington, VT, 05405, USA; 2 Biochemistry, University of Vermont, Burlington, VT, 05405, USA

**Keywords:** Peer Teaching, Cadaver Donors, Medical Students, Anatomy, Medical Education

## Abstract

**Background: **Peer teaching is a powerful educational tool utilized in medical school curricula. Previously, first year medical students taught their peers about the gross anatomical structures they had dissected in the anatomy lab. While this strategy provided an opportunity for students to learn from one another, there were unintended outcomes including difficulty engaging all students. Considering these observations, along with needing to limit student numbers in the lab due to the coronavirus disease 2019 (COVID-19) pandemic, a strategy was developed where students could conduct their anatomy peer teaching in a virtual environment. The goal was to establish an effective and efficient means for students to teach and learn from one another virtually.

**Methods: **Students, working in groups of four, were tasked to: 1) Find and label 4-5 assigned structures on cadaver-based images; 2) Provide a rationale for labeling; 3) Discuss something relevant about the structure; 4) Prepare a 5-minute video presentation of steps 1-3; and 5) Review and provide meaningful feedback on another group’s presentation. Student performance on virtual peer teaching assignments was evaluated using a structured rubric and grades were weighted based on two separate faculty assessments.  Student feedback was obtained via discussions with the course director, a semi-structured 1-hour virtual focus interview and from course evaluation data.

**Results: **While students performed well on these assignments, feedback from students indicated several drawbacks such as excess time editing their videos, concerns about the validity of information provided by their peers, and the timing of peer teaching to be non-conducive to learning.

**Conclusions: **Although the students viewed the virtual peer teaching negatively, we were successful in developing a platform in which students participated more equally in peer teaching. Recommendations to those considering this platform include careful consideration of timing of peer teaching activities and faculty feedback as well as technology used.

## Introduction

Peer-assisted learning (PAL) is defined as “the development of knowledge and skills through explicit active helping and supporting among status equals or matched companions, with the deliberate intent to help others with their learning goals” ( Topping, 2008). As such, PAL has been reported to encompass three modalities of peer education including peer mentoring, peer tutoring and peer teaching. With respect to peer teaching, the term is broadly defined as one student teacher instructing another student or a group of fellow students ( Ten Cate & Durning, 2007). Now an integral component of medical education, several reports have documented the numerous benefits of peer teaching on peer teachers and learners as well as the faculty. For the peer teacher, students and faculty have reported that peer teaching supports better communication, development of organization and leadership skills, and helps the student teacher better retain and apply their knowledge ( Agius *et al.,* 2018; Allikmets & Vink, 2016; AlShareef *et al.*, 2019; Bentley & Hill, 2009; Dunham, 2018; Han *et al.*, 2015; Krych *et al.,* 2005; Manyama *et al.*, 2016; Newman, 2021; Youdas *et al.,*, 2008) as well as give and receive feedback ( Cushing *et al.*, 2011). Interestingly, Agius *et al.* (2018) reported both short-term and long-term benefits of peer teachers in an undergraduate medical gross anatomy course, reporting that peer teachers performed better on the subject they had taught as well as anatomy exams administered after the PAL experience ended. For peer learners, peer teaching can improve performance on examinations because of better understanding and retention of materials ( Manyama *et al.*, 2016) as well as support self-regulated learning ( Alzaabi *et al.*, 2021). Peer learners also feel more at ease discussing the material with their peers, a finding which may be attributed to peers being more approachable and understanding of where the students are in their learning ( AlShareef *et al.*, 2019; Khapre *et al.*, 2021; Lockspeiser *et al.*, 2008). For faculty members, peer teaching can alleviate the burden of instruction, especially in demanding courses such as anatomical dissection laboratories ( Bentley & Hill, 2009; Han *et al.*, 2015; Manyama *et al.*, 2016).

Peer teaching has been classified by ( Ten Cate & Durning, 2007) as teaching that occurs from students who are more advanced in the curriculum (near-peer teaching) and teaching that occurs from students who are at the same stage in their academic training (peer-peer teaching) ( Ten Cate & Durning, 2007). As an example of near-peer teaching in gross anatomy, Youdas *et al.* (2008) reported that first-year physical therapy peer learners indicated a better understanding of anatomical relationships as a result of teaching materials prepared by second-year physical therapy peer teachers. A sub-category of peer-peer teaching is reciprocal peer teaching (RPT) in which the students alternate at teachers and learners ( Krych *et al.,* 2005; Topping, 1996; Whitman & Fife, 1988). All aforementioned forms of peer teaching have been employed in a number of settings in the medical curriculum including summative exams, diagnostic imaging, clinical skills assessment, problem-based learning, tutorials on subject matter areas and gross anatomy ( Engels *et al.*, 2018).

Within the gross anatomy lab, RPT has been extensively used to support student self-directed learning of anatomy, understanding of anatomical concepts, communication skills and teaching effectiveness. Krych *et al.* (2005) reported that RPT improved student communication skills and understanding of the anatomical content that students taught, citing that the majority of student teachers perceived they had gained valuable interpersonal skills that would translate to their careers as physicians. Bentley & Hill, (2009) used RPT to assess the effects of this approach on student knowledge of anatomy. The authors found that while RPT did not improve student performance on practical exams when compared to classes which did not engage in RPT, it was overall positively accepted by the students who indicated that it improved their understanding of the material ( Bentley & Hill, 2009). Similarly, Dunham 2018 demonstrated no significant differences in practical exam and lecture exam grades following the introduction of RPT into the medical anatomy course at the Indiana University School of Medicine when compared to prior cohorts of students who did not participate in RPT, but did report improvements in student and instructor satisfaction with the intervention. Interestingly, the author reported that students who actively engaged in the dissection and in turn, taught their dissected structures to their peers, were more likely to correctly answer practical exam questions on that content, a result the author acknowledged may result from dissecting the structures, teaching the dissected structures or both ( Dunham, 2018).

Some studies have demonstrated a positive correlation between the introduction of RPT and student academic performance in gross anatomy. In an earlier study, for example, one quarter of the students taking a medical gross anatomy course at St. Louis University in Missouri were given a staff-directed demonstration of a pro-sected cadaver and went on to teach this material to their peers ( Yeager & Young, 1992). In the end, all students participated in RPT and the authors reported that in addition to fewer cadavers needed and less faculty time devoted to instruction, that students performed better than the national average on the anatomy part of the National Board Examination ( Yeager & Young, 1992). Using a Likert scale to assess student teacher and learner opinions regarding anatomical knowledge and understanding, Krych *et al.* (2005) found that the majority of student teachers who engaged in RPT in the gross anatomy lab, believed that their role as a teacher promoted better retention of the materials as opposed to the majority of student learners who did not have this opinion. A study which built upon the work of Dunham (2018) reported that, as a result of the incorporation of RPT in the medical gross anatomy course at the Indiana University School of Medicine, student teachers were more likely to correctly answer exam questions in comparison to peer learners ( Newman, 2021). Despite this benefit, evaluation of recorded peer teaching encounters demonstrated that peer teachers only used on average about half of the time allocated to peer teaching, resulting in diminished teaching quality ( Newman, 2021). Manyama *et al.* (2016) found that use of RPT in the gross anatomy lab improved student teacher preparedness for dissections and confidence for teaching the material, demonstrating that the introduction of RPT resulted in significantly higher student grades in the course than those obtained prior to RPT. Finally, ( Han *et al.*, 2015) employed a unique peer teaching strategy in which first-year medical students were separated into experimental groups who received guidance on the dissection from peer teachers and a control group who received guidance on the dissection from a faculty member. Students in the experimental group led by the peer teacher rated their knowledge of the upper limb anatomy better and had significantly higher exam scores on the upper limb content than those students led by a faculty member ( Han *et al.*, 2015). These and other studies support the positive impact that peer teaching in the anatomy lab can have on students’ performance and perception of their knowledge.

**Figure 1.**
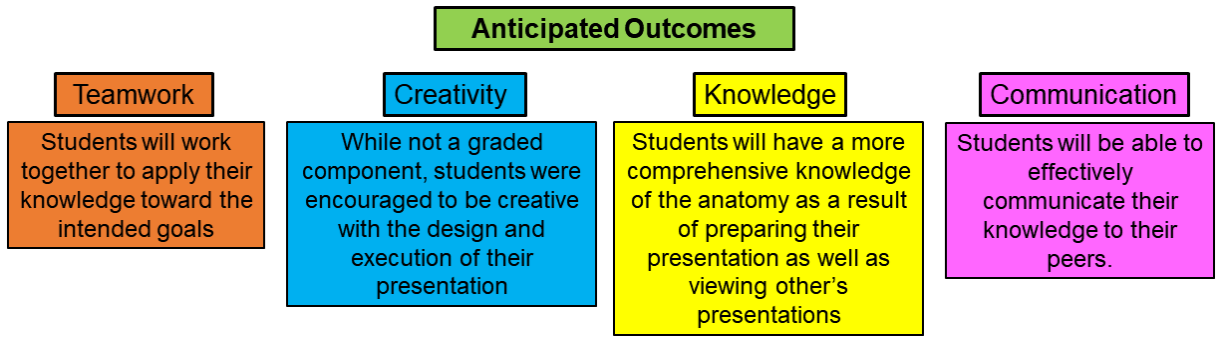
Anticipated learning outcomes for student teachers and learners.

At the Larner College of Medicine (LCOM), half of the class of first-year medical students have historically alternated between teachers and learners in the anatomy lab and thus engaged in RPT. While this strategy enabled direct peer-peer instruction, the major drawback was that not all students were able to engage in peer teaching activities. Often, there were up to six members present at each table, causing crowding and high noise, which limited student participation in the activity. In light of this limitation, coupled with the need to move to a partially remote learning environment as a result of the coronavirus disease 2019 (COVID-19) pandemic, we transitioned the anatomy peer teaching to a virtual platform. Using this virtual approach to peer teaching, it was expected that first-year medical students would gain: 1) team working skills through collaborating with other members of their group to build and deliver their presentation, 2) knowledge about the subject through preparing the materials as well as viewing materials provided by their peers, and 3) communication skills through effectively presenting the content in a logical and well-organized manner ( Figure 1). While not graded, it was anticipated that students would also benefit from employing creativity to design their presentations. In this study, quantitative data was collected on practical exam performance and faculty assessments of peer teaching materials while a thematic analysis was used to evaluate student perceptions about the peer teaching approach following a focus group session and from student feedback in the course evaluation.

## Methods

### Ethics statement

According to the policy defining research activities which constitute research at the University of Vermont/University of Vermont Medical Center, this work met criteria for operational improvement activities exempt from ethics review. For the focus group interview (described later), all first-year medical students received an email invitation to participate in the voluntary discussion. Details regarding the number of student volunteers sought, facilitator, purpose, structure and timing of the discussion were provided in the email correspondence with instructions for any interested students to submit questions and intent to participate to the facilitator. The focus group was conducted after course grades were final and focus group participants were informed the course faculty would not have access to identifiable data from the focus group.

### Participants and virtual peer teaching assignments

**Figure 2.**
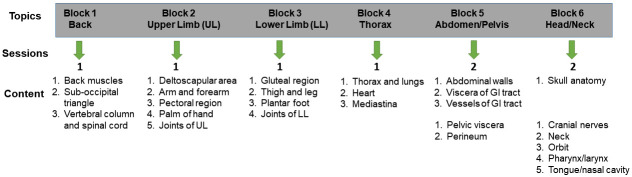
Anatomy content and associated virtual peer teaching topics.

A total of 124 medical students matriculated into the medical program at LCOM in 2020 (e.g. Class of 2024). The curriculum for the class of 2024 was conducted in a hybrid manner allowing students to participate in anatomy lab dissections only on their assigned on-campus days. Students were divided into 4 lab groups, with each group having 31 students. These groups typically participated in 1 in-person lab dissection per Block ( Figure 2).

One member from each of the four lab groups was placed in another cohort of students (peer teaching group, N=31) who prepared virtual anatomy teaching materials for their peers. Students were given a total of eight virtual peer teaching assignments which corresponded to content in their anatomy Blocks ( Figure 2). In some Blocks, such as the Abdomen/Pelvis and Head and Neck, there were two virtual peer teaching assignments ( Figure 2). The timing of most peer teaching activities came toward the end of a particular Block so students had time to complete the dissections and review the material.

### General course details

The anatomy curriculum at the LCOM is part of a larger 18-week course: Foundations of Clinical Sciences (FoCS), which, introduces students to many disciplines in the basic sciences including anatomy, physiology, embryology, cell biology, imaging, metabolism, histology, genetics and ethics and doctoring skills. Most disciplines of FoCS are taught longitudinally during the 18-week course and where appropriate, were introduced by region of the body (e.g. Upper Limb). For instance, students learned the gross anatomy of the abdomen along with the relevant imaging, physiology and histology of the abdomen.

The anatomy portion of the course encompasses all 18 weeks of FoCS and is broken up into Blocks focused on regional anatomy as shown below and additionally illustrated in Figure 2.

Block 1: Back. Students dissect the superficial, intermediate and deep back muscles and perform a laminectomy to harvest the spinal cord.

Block 2: Upper limb. Students dissect the scapular and pectoral regions along with the arm, forearm and hand (dorsum and palm of hand). Students are given the opportunity to dissect joints of the upper limb if they wish to do so, but are provided with several pro-sections of the shoulder, elbow and wrist to supplement their knowledge of the anatomy.

Block 3: Lower limb. Students dissect the anterior and medial thigh, gluteal region and posterior thigh, posterior leg and popliteal fossa, anterior and lateral leg and the foot (dorsum and plantar aspects). Students are given the opportunity to dissect the joints of the lower limb if they wish to do so, but are provided with several prosections of the hip, knee and ankle joints to supplement their knowledge of the anatomy.

Block 4: Thorax. Students dissect the anterior thoracic wall, lungs, heart and superior and posterior mediastina.

Block 5: Abdomen/pelvis. Students dissect the anterior abdominal wall and subsequently dissect the vessels of the celiac trunk and the superior and inferior mesenteric arteries before removing the contents of the GI tract. Removal of the GI tract facilitates dissection of the posterior abdominal wall. Students then proceed to hemisect the pelvis to learn the pelvic anatomy and pelvic neurovasculature.

Block 6: Head/neck: Faculty remove the calvaria and brains to give students a guided tour of cranial nerve anatomy. Students then dissect the triangles and root of the neck before proceeding to dissect the orbit from the anterior and superior approaches. Students then review the anatomy of the posterior pharynx and larynx before performing a head bisection to review the anatomy of the nasal and oral cavities.

Since 2006, students have traditionally been placed into two dissection groups (Groups A and B), resulting in a total of 6-8 students assigned to one of twenty donors in the lab. A typical lab session involved 3–4 students in Group A performing the assigned dissection. In the next lab session, 3–4 students in Group B met with members of Group A to learn the anatomical structures Group A dissected. This alternated for the following lab session where the members of Group A met with members of Group B to learn about the structures they dissected. In this manner, students participated in RPT from 2006 to 2020 after which instruction was largely virtual and peer teaching was also shifted to a virtual platform.

Prior to 2020, assessments included practical laboratory exams which occurred for each block of anatomy. Here, anatomical structures were tagged on each donor and/or pro-sections available in the lab and students were asked to (A) identify the structure and (B) describe something about the structure. Typical part B questions asked about innervations, muscle attachments, blood supply, etc. In addition, students were tested on their anatomical knowledge in written exams. Questions on written exams were often application-based, assessing students’ ability to apply their anatomical knowledge to a clinical scenario. Since students did not participate in in-person examinations in 2020, their practical exams were conducted virtually using cadaveric images as opposed to cadaveric donors. The structure of the exam was similar in that students were asked to (A) identify the structure and (B) describe something about the structure.

With the exception of the Abdomen/Pelvis and the Head/Neck Blocks which had two peer teaching assignments, there was one peer teaching assignment per block. The topics that were covered in the peer teaching assignments are also shown in Figure 2.

### Student expectations for virtual peer teaching

To prepare virtual anatomy peer teaching materials, students were given online access to a PowerPoint ^®^ containing donor images associated with that Block’s material. The donor images were obtained from Anatomy: A Photographic Atlas (Rohen *et al.*, 8 ^th^ ed.) and were utilized with permission from and a usage fee payment to the publisher, Wolter’s Kluwer. Access to the images were limited to students having LCOM credentials.

All 31 peer teaching groups participated in preparing 1-2 peer teaching activities/block. Each student group was given 4–5 anatomical structures/peer teaching assignment to identify and label on the provided donor images. The assignments consisted of anatomical structures corresponding to a given body region ( Figure 2). For example, in Block 2 (Upper Limb), students were assigned structures that corresponded to the shoulder and pectoral region, arm, anterior forearm, posterior forearm and dorsum of hand, palm of the hand and joints of the upper limb. Structures were assigned in such a way so as to minimize overlap between groups. This was intentional so that students could reference and learn different content from their peers. Additionally, the authors attempted to allocate structure assignments in such as a way so that student groups had to demonstrate a variety of structures. For the Upper Limb, for example, all student groups except those assigned with the joints, were asked to demonstrate some combination of muscles, nerves and vessels. When preparing materials, student peer teaching groups were given the following objectives:

*1. Select the best images from the provided image set and label these based on your structure list, noting anatomical relationships and key anatomical information.*

*2. Prepare a short (~5 minute) presentation (6–12 slides) of your work using Camtasia* ^®^

*a.*  *Discuss how you identified the structures using anatomical relationships*
*i.*  *Was this the most superficial structure, was it innervating X muscle, etc.*

*b.*  *Discuss one relevant bit of information about each structure assigned*
*i.*  *Action, attachment, innervation, clinical deficit if injured.*

*c.*  *Upload the video to LCOM FoCS 2024 Microsoft Teams* ^®^ *Block folder before the video due date listed provided in the syllabus*.

*3. Review another group’s presentation and provide meaningful feedback*

Each student was asked to review another group’s presentation. Here, students were given the flexibility to review any video they wished and then provide meaningful feedback to the student peer group who had prepared the video. Each student reviewed and provided feedback for 1–2 presentations/Block for a total of 8 during the course.

**Figure 3.**
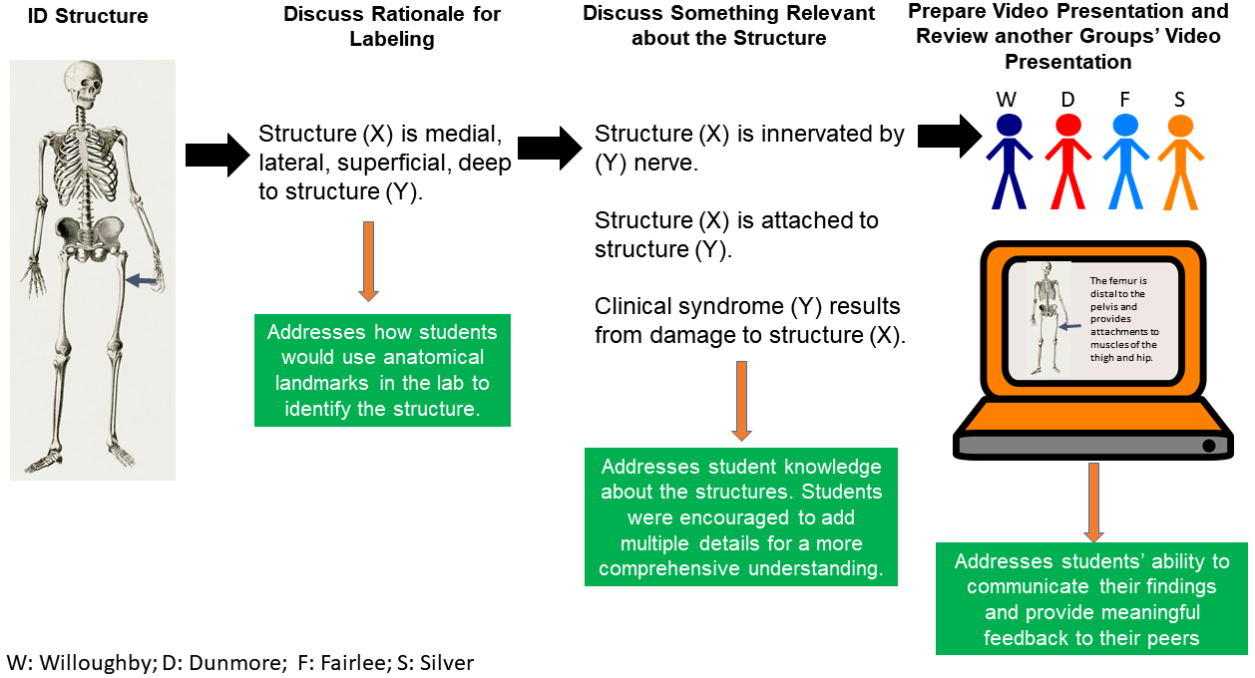
Goals for virtual peer teaching activities. Students, working in groups of four, were given a list of structures to label on provided cadaveric images. Students were tasked with discussing the rationale for labeling in addition to discussing one relevant piece of information about each structure. Students prepared a video presentation using Camtasia, uploaded the video for their peers to view and additionally viewed and provided feedback to another group’s video.

Refer to Figure 3 for a visual summary of the peer teaching objectives. Students were given an instructional document as well as a virtual synchronous tutorial on use of Camtasia® for video creation and editing. In general, *Camtasia*® 2020 (TechSmith, East Lansing, MI) is a software program which enables one to record content on their desktop, laptop or tablet screen. The presenter is also able to record audio and visual elements as they proceed through the presentation. Once the recording is completed, students were able to access and review the recording in *Camtasia*®. Student authors had the option to edit their presentation, including addition of annotations, animations and audio/visual effects as well as removing content that the authors deemed unnecessary or erroneous. All peer teaching recordings were uploaded by the student presenter as MP4 files into a specific folder in Microsoft Teams®. Seven percent of the students’ scores in the course was allotted to virtual peer teaching presentations.

### Student instructions for providing feedback

Each student was responsible for reviewing another peer teaching groups’ presentation and providing feedback to those members. Students were instructed to provide feedback that was meaningful and helpful for the group and were asked to view content from a lab region that they weren’t assigned. Students were instructed to consider the following when providing feedback:

1. Did the group miss something important about the structure(s)?

2. Was something reported incorrectly? Structures improperly labeled or discussed.

3. Were components of the presentation difficult to hear, visualize or follow?

Students provided anonymous feedback to their peers using Blackboard and the course director oversaw comments. If a student’s feedback was deemed to lack specificity (e.g. “Nice presentation,” “Looks good,” “Nothing to comment on,” etc.), the student was contacted by the course director who instructed the student on strategies to improve their feedback. A total of 0.5% of the student’s grade in the course was allotted to peer feedback.

### Faculty grading of student presentations

Student groups were given a compiled grade on their presentation based on two faculty scores. A sample grading rubric is provided in the *Extended data* ( Hielscher, 2022b). The grading strategy was weighted in such a way that 75% of the grade was based on the accuracy of information and 25% of grade was based on features of the presentation. If any component of faculty feedback appeared contradictory, the anatomy director reviewed the presentation and faculty comments and made a recommendation to the course director about the most appropriate feedback and grade to provide the students.

Students utilized this method of creating peer teaching videos from Blocks 1 – 4 at which point it was elected to switch from the use of Camtasia ^®^ to PowerPoint ^®^. All aforementioned methods were utilized, but the grading rubric was changed to reflect the use of PowerPoint ^®^. Here, 84% of the grade was given to accuracy of information while 16% of the grade was devoted to visual and organizational features of the presentation ( *Extended data,* Hielscher, 2022b).

### Data collection

Student peer teaching group scores corresponding to the eight virtual peer teaching assignments were collected by the course director.

At the conclusion of the course, student feedback about the perceived usefulness of the virtual peer teaching platform was sought. To solicit student involvement, an email announcement was sent to the first-year medical students inviting their voluntary participation in a 1-hour semi-structured focus group. A total of six first-year medical students volunteered to participate in a virtual (Zoom) focus group, which was led by an experienced qualitative researcher not affiliated with the course. The researcher used an interview guide designed by the authors who sought to determine whether student preparation and/or review of virtual peer teaching presentations was effective at enhancing their anatomical knowledge. Student comments were recorded, transcribed and analyzed as described below. In addition to the focus group, students were also queried using a Likert Scale to rate how often they used particular resources provided in the anatomy curriculum. All 124 first-year medical students were given the opportunity to rate the anatomical resources and materials used in the course. The students provided this feedback as part of their course evaluation. There were many anatomy resources available to students of which one was the virtual peer teaching ( Table 1).

**Table 1.** Student use of anatomy resources. **Anatomy E-modules:** faculty pre-recorded materials on anatomical theory. **Atlas:** Grant’s, Netter’s, etc. **Clinical Correlations Sessions:** faculty-led question-based sessions intended to integrate anatomical concepts. **Complete Anatomy Software:** 3D anatomy educational tool from 3D4Medical. **Anatomy Dissection Videos:** faculty-prepared videos on dissected anatomical structures. **Faculty Donor Review Sessions:** faculty-led review sessions on cadaveric images. **Laboratory Checklists:** structures that students were responsible for identifying on the donors and donor images. **Laboratory Dissections:** in-person assigned laboratory dissections. **Donor Anatomical Images:** images students used for preparing peer teaching presentations. **Peer Teaching Presentations:** virtual peer teaching materials. **Faculty Q and A Sessions:** weekly forums for students to ask faculty anatomy questions. **Worksheets:** fill-in-the blank and structure identification documents for each laboratory session

Resource	Used Frequently	Used Occasionally	Used rarely	Never used	Number
**Anatomy E-Modules**	86	29	4	4	123
**Atlas**	42	31	24	24	121
**Clinical Correlations Sessions**	104	14	4	1	123
**Complete Anatomy Software**	25	24	48	25	122
**Anatomy Dissection Videos**	56	38	27	2	123
**Faculty Donor Review Sessions**	105	10	4	4	123
**Laboratory Checklists**	76	34	11	3	124
**Laboratory Dissections**	72	27	21	2	122
**Donor Anatomical Images**	57	29	18	19	123
**Peer Teaching Presentations**	7	13	33	70	123
**Faculty Q and A Sessions**	27	14	33	50	124
**Worksheets**	48	25	28	23	124

### Thematic analysis of focus group comments

A six-step thematic analysis method, which includes 1. familiarization with the data, 2. generating initial codes, 3. searching for themes, 4. reviewing themes, 5. defining and naming themes, and 6. producing the report, was used.; ( Braun & Clarke, 2006; Coates *et al.*, 2021; Kiger & Varpio, 2020). Examination of coded data led to identifying, defining and naming of themes. The qualitative researcher used an inductive approach to data analysis, working from a constructivist approach Coding was done using Microsoft Word. Saturation of ideas, indicative of no additional categorical data obtained ( Glaser & Strauss, 1967), was reached within the session. Course evaluation free text comments offered an additional source of data to the investigators; this data triangulation contributed to the trustworthiness of the findings.

Thematic analysis, a method to analyze qualitative data for the purpose of identifying and reporting common themes ( Braun & Clarke, 2006), was employed because of its utility to help explore and understand experiences and shared meanings which aligned with the goal of this focus group ( Kiger & Varpiro, 2020). As such, thematic analysis of transcribed comments made from first-year medical students during the focus group discussion was conducted by the focus group facilitator/qualitative researcher to better understand student perceptions on the effectiveness of virtual peer teaching as an educational tool for learning the anatomy. In this study, thematic analysis was used within a constructivist approach to the work. This means the thematic analysis was used to “emphasize the social, cultural, and structural contexts that influence individual experiences, enabling the development of knowledge that is constructed through interactions between the researcher and research participants, revealing meanings that are socially constructed” ( Kiger and Varpio, 2020, p. 2). A related method for analysis of qualitative data is grounded theory, which is classified as a positivist approach in that it seeks to test hypotheses and generate theories to explain the researcher’s findings ( Braun & Clarke, 2006; Eisenhardt, 1989).

A six-step thematic analysis method, which includes: 1) familiarization with the data, 2) generating initial codes, 3) searching for themes, 4) reviewing themes, 5) defining and naming themes, and 6) producing the report, was used ( Braun & Clarke, 2006; Coates *et al.*, 2021; Kiger & Varpio, 2020). Examination of coded data led to identifying, defining and naming of themes. The qualitative researcher used an inductive approach to data analysis, working from a constructivist approach Saturation of ideas, indicative of no additional categorical data obtained ( Glaser & Strauss, 1967), was reached within the session. Collected data was maintained in Microsoft Word (Microsoft Corporation, Redmond, WA). Course evaluation free text comments offered an additional source of data to the investigators; this data triangulation contributed to the trustworthiness of the findings. Reflexivity was considered in the data analysis. Two of the authors (AH and SE) are faculty in the course and while they played major roles in designing the virtual peer teaching platform, they did not participate in focus group interviews or data analysis. Additionally, the qualitative consultant (KH), is assistant dean for medical education and a social scientist whose work is informed by her constructivist perspective on learning, i.e., the perspective that individuals “construct new knowledge through the interaction between their previous skills and knowledge, the skills and knowledge gained from social interaction with peers and teachers, and social activities” ( Mukhalalati & Tayor, 2019, p. 7). Although not an anatomist, she is knowledgeable about the medical school curriculum.

### Data analysis

A grade for each peer teaching assignment was given by the course director and was made based on the weighted averages given by two faculty members. The only peer teaching assignment which was not included in the students’ grade for the course was the first one (Block 1). Average scores for each peer teaching assignment in addition to students’ preferred anatomy resources were graphed and analyzed using Graph Pad Prism v9 for Windows (GraphPad Prism Software, San Diego, CA) (RRID:SCR_002798). Results are reported as the means ± standard deviation (SD). An alternative to Graph Pad Prism is Excel. Course evaluation data containing student comments on peer teaching as a resource were analyzed for themes using a frequency count and were categorized accordingly. The frequency analysis was completed by AH.

## Results

### Student performance on virtual peer teaching activities

**Figure 4.**
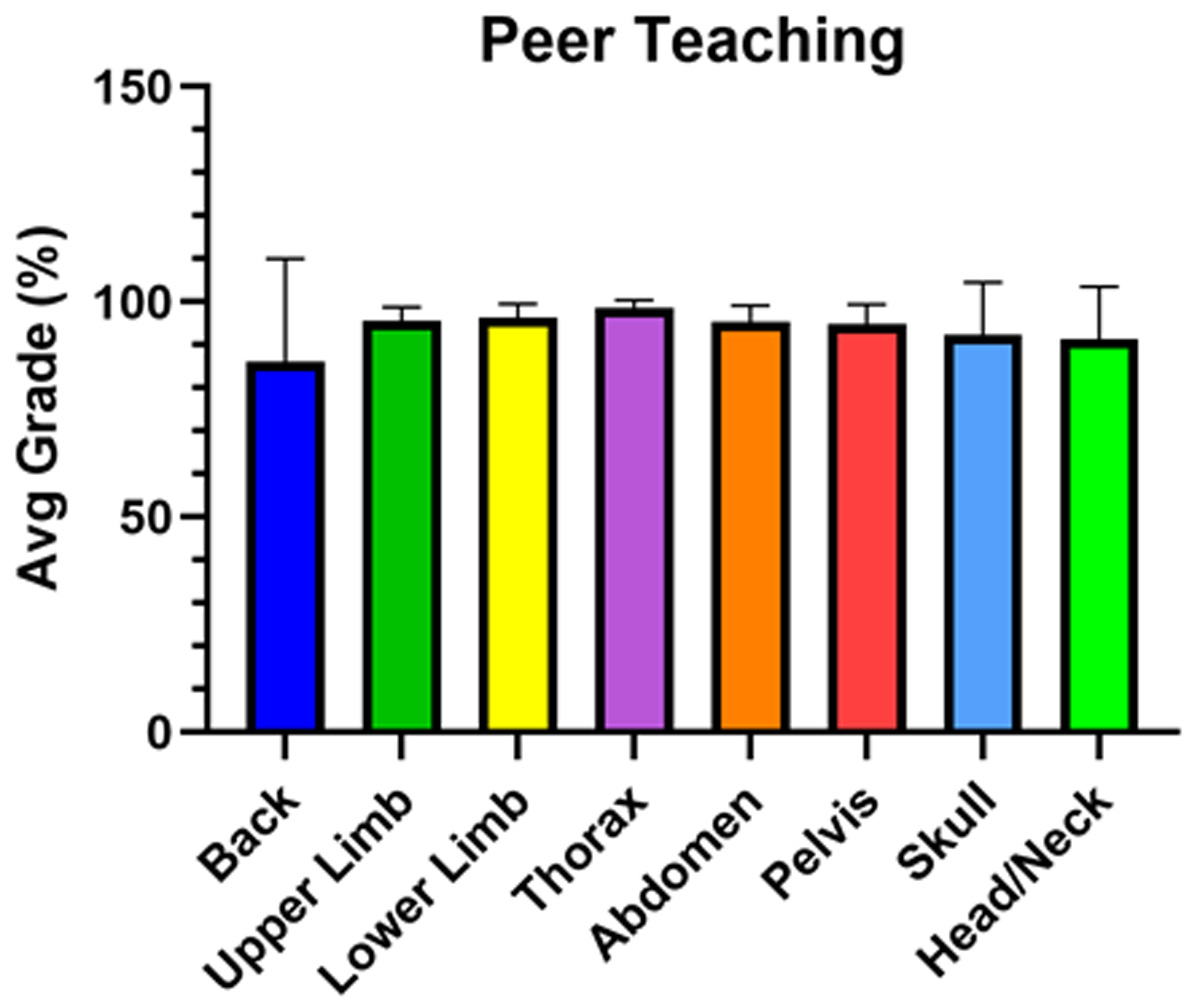
Average grades for each of the peer teaching activities. Overall, students performed well on each virtual peer teaching activity as assessed by two faculty members and averaged for all 31 peer teaching groups. The only virtual peer teaching activity that was not included in the student’s final grade was the back (Block 1).

In this study, 1 student from each of the 4 lab dissection groups was placed in a virtual peer teaching group (4 members/group). With a total of 31 peer teaching groups, this strategy was intended to encourage a more collaborative approach as groups consisted of small numbers of students. Furthermore, members represented each of the dissection groups and having participated in 1-2 dissections/Block, could thus provide a unique perspective about the labeling of structures relevant to their assigned dissection. Overall, students performed well on each of the virtual peer teaching assignments ( Figure 4). The first assignment on the back (Block 1), although graded, was not included in the students’ final grade. The scores for each peer teaching assignment reflect the average of grades for all 31 peer teaching student groups ( Figure 4) ( Hielscher, 2022a).

### Student feedback to peers

Students engaged in peer feedback for each of the eight virtual peer teaching assignments. Overall, the majority of students provided feedback that was meaningful. An example of meaningful, constructive student feedback from the upper limb (Block 2) is shown below:

*“The overall quality of the video was great! I enjoyed the pace, it let me follow along and learn in an appropriate pace. There were great pauses for me to absorb the information. My only feedback would be the amount of text on the slides. I wish it was just the pictures and maybe clinical relevance because you explain everything so well. Therefore, I think having all the text almost hinders the image-- it makes the image smaller and a bit harder to appreciate. That being said the clinical relevance portion was so cool and really helped me fully integrate things. All in all, great job! **”***

### Student perceptions on virtual peer teaching activities

Toward the later part of the course (Block 5), student feedback to the course director indicated that the majority of the class was in favor of moving from recorded presentations in Camtasia ^®^ to PowerPoint ^®^ presentations. Here, students informed the course director that they were spending too much time editing their videos to ensure accuracy and flow of information, noting this time was spent inefficiently. The change was supported by the faculty and course director and while the expectations remained the same, the grading rubric was modified as described in the *Methods* section and provided in the *Extended data* ( Hielscher, 2022b).

At the end of the course, student feedback was solicited on the perceived usefulness of peer teaching as a means to improve anatomical knowledge. Student feedback was requested in two ways. First, students were asked in the course evaluation to rate how often they utilized anatomy resources to facilitate their learning. Of the 12 anatomical resources available, students rated virtual peer teaching to be the least used ( Table 1). The most utilized resources were faculty led donor image review sessions and clinical correlations sessions ( Table 1). For the faculty led donor image reviews, the anatomy faculty virtually guided students through the process of identifying anatomical structures, discussed relationships and answered student questions in real time. For the clinical correlations sessions, faculty led students through solving clinically-oriented questions, intended to integrate anatomical concepts. Students were given the opportunity to comment on the anatomy resources. Of the 124 comments, 21 student comments (17%) dealt with virtual peer teaching. Students frequently cited virtual peer teaching to be an ineffective study tool, often commenting that it was “busy work,” a “waste of time,” and “time-consuming.”

Second, student volunteers in the focus group discussed the benefits and drawbacks of virtual peer teaching assignments. Six major themes were identified.

*The timing of peer teaching activities came too late in the Block.* A consequence of placing peer teaching assignments at a later point in the Block was that students did not find them useful for studying. As one student commented “They didn't give feedback to us until we were done with our last set of PowerPoint. So I personally didn't review any of the feedback I received. And so I didn't make any adjustments to how we were doing it.” (S2)

*Valuable peer teaching was happening in other places.* Students were engaging in meaningful peer teaching activities during assigned laboratory times as well as in real-time virtual formats. Students cited peer-generated quizzes as one example “So regardless those peer created teaching or exams were super helpful and I think probably 95 to 98% of the class use that just for our studying purposes.” (S4)

*Faculty expertise was valued.* The materials provided by peers were perceived to be less trustworthy than that provided or at least reviewed by faculty members. For example, one student noted “And then it obviously takes the anatomist some time to like go through and look at what we've done and make sure that what we've done is correct. So like, I wouldn't really want to be using, I guess, the PowerPoints and stuff as my study tool.” (S5)

*Technology was difficult to use.* The technology (Camtasia® and Microsoft Teams®) was cited to be cumbersome and time-consuming. Specifically, students mentioned that groups were spending hours editing recordings. Multiple platforms also decreased efficiency as explained by this student “Much of our work with this peer teaching stuff, we had to go on Microsoft teams and though we eventually figured out how to submit things and upload things and stuff. I mean, it's, just another technological modality that kind of complicates the entire process.” (S4)

*Assignment weight had mixed perspectives on motivation.* Some students believed that there was too much weight while others believed there was not enough weight on the peer teaching assignments. For example, one student commented “Like for example, it was a lot of work for 1%...I actually would have paid more attention to it just because it had been worth more.” (S1)

*Faculty feedback on the students’ work was valued.* Students perceived that the consistency and quality of faculty feedback decreased over time, a point that the students believed to result from increased demands on faculty time.

In addition, there were two minor themes identified in the focus group:

*Faculty demonstrated concern for learning and Challenge level impacted perceived value.* For the latter, participants shared that if an assignment was perceived as less challenging it made it difficult for students to offer critique and this influenced their perception of the experience. For example, one student shared “I feel like people were just like picking, like, like they were just coming up with like, with a weirdest feedback to try to get the points.” (S5)

## Discussion

The key findings of this study demonstrate that first-year medical students did not view the virtual anatomy peer teaching as implemented in 2020 to be a useful tool to support their learning of anatomy. While this peer teaching strategy was implemented in light of COVID-19, it was anticipated that this approach would address some of the challenges that had historically surrounded in-person peer teaching, such as difficulty engaging all students due to crowding and high noise levels in the lab. Indeed, students were more engaged in peer teaching as demonstrated from student participation and their preparation of high-quality teaching materials. The majority of students also provided their peers with constructive, meaningful feedback. It is also worth mentioning that in spite of the unique nature in which content was delivered, that overall student performance in anatomy did not significantly differ between years 2020 (hybrid delivery) and 2019 (in-person). Despite these positive outcomes, student input indicated that virtual peer teaching was time consuming and did not improve their overall knowledge of anatomy.

One of the major concerns that students first addressed was the difficult and time-consuming nature of using Camtasia ^®^ to generate video presentations. While the faculty provided written instructions and a live virtual demonstration on how to use Camtasia ^®^, they never intended for students to perfect their recordings and in this regard, better communication may have contributed to less student time devoted to editing. In light of the challenges surrounding the use of Camtasia ^®^, students were allowed to use PowerPoint ^®^ in place of video recordings, a move that was made toward the end of the course at Block 5. In retrospect, a better method may have been a recorded how-to video which provided a detailed step-by-step instructional guide for the students to reference during preparation of teaching videos using *Camtasia*®.

Another common concern amongst students who participated in the focus group as well as from comments associated with the end of the course survey was that they perceived the virtual peer teaching to be busy work. In part, this could be due to the use of alternative peer teaching methods or learning strategies that the students had developed during the course. For example, students cited that meaningful peer teaching was occurring in live Zoom sessions with their peers. Other students noted that peer teaching was occurring in the anatomy lab during assigned dissection times. Interestingly, the majority of students cited that the most valuable resource for learning the anatomy came from faculty-led review sessions. Faculty-led donor reviews were not originally planned in the curriculum and were added early on in the course to assist students with their anatomical learning. The authors had originally anticipated that student peer teaching materials would have served this goal. While there are multiple reasons for students to view faculty led sessions more favorably, one reason may be that students felt they could not trust the information provided by their peers. Indeed, ( Bentley & Hill, 2009) reported that first-year medical students cited inadequate instruction from their peers during RPT sessions in the gross anatomy lab. Similarly, others have also cited student perceived inadequacies of learning from their peers in the gross anatomy lab ( Dunham, 2018; Manyama *et al.,* 2016; Newman, 2021). Interestingly, Newman (2021) indicated that medical student responses illustrated a preference for the role of peer teacher as opposed to the role of the peer learner, as peer learners were more likely to distrust the information provided by the peer teachers. While students may have a natural inclination to view information from faculty as being more trustworthy, it is possible that certain measures may have reduced this perception. For instance, had faculty vetted peer teaching materials earlier in the Block, students may have had a different perception on the trustworthiness of their peers’ materials and would have been more inclined to use these as a study resource.

 Another reason which may have led to the lack of student satisfaction with the virtual peer teaching was the asynchronous nature by which materials were developed and reviewed. When students previously engaged in in-person peer teaching, they were able to provide and receive dynamic feedback from their peers and faculty, enabling them to correct any misconceptions and validate their knowledge in the moment. The opportunity for students to interact with one another and with faculty can be highly rewarding and motivating for students while also providing a more humanistic touch. Thus, an important feature of feedback is that it must be easily understood and interpreted by the student ( Henderson *et al.*, 2019). To construct narrative feedback that is free from misinterpretation is challenging to create, even when following a provided rubric. One way that others have circumvented this setback in asynchronous learning environments is through the use of audio recorded feedback. For example, ( Ice *et al.*, 2007) analyzed graduate student interview responses regarding the use of audio as opposed to text-based feedback and reported that the use of audio recorded feedback supported higher student satisfaction, conveyed nuances better, increased students’ sense of involvement in the course, and supported better retention of material ( Ice *et al.*, 2007). The human quality that comes with an audio recording was portrayed to reflect a sense of caring and social presence by the instructor ( Ice *et al.*, 2007). In the present study, it is possible that student perceptions on the virtual peer teaching exercises may have been different had the faculty and peer reviewers provided audio recordings of their feedback, improving student satisfaction and motivation. While this modality of feedback may have improved student motivation, the content of the feedback is equally if not more important. Here, feedback that it is constructive, allowing the learner to identify weaknesses and improve upon these, is most supportive for learner development. One of the themes from the focus group was that students perceived that the quality of faculty feedback decreased over time, which likely played a role in decreased student motivation for preparing peer teaching materials. Tuma and Nassar (2022) described that many educators providing feedback do not give specific details, utilizing generalities including “good job” or “well-done,” statements which provide little educational value to the recipients. The authors emphasize that learners must understand why components of their performance were done well or poorly and in turn, given guidance on what steps they can take to improve their weaknesses ( Tuma & Nassar, 2022). Taken together, giving students audio-based feedback to add a humanistic touch as well as providing students with consistent, timely, and constructive feedback would have likely been key elements for engaging student development in their virtual learning environment.

While several studies have documented the benefits that peer teaching can have on student teachers and learners and faculty within the medical curriculum, there are many challenges that remain. One challenge is to find the best method of introducing it into the curriculum. As many medical schools differ in the way in which content is delivered in the curriculum as well as considerations of space, availability of faculty and numbers of students, the implementation of peer teaching is likely driven by institutional and curricular needs and a one-size fits all model likely will not work for all medical schools. While the authors attempted to implement a strategy that could foreseeably be applied in multiple educational settings given the virtual nature, there were limitations as discussed in this report as well as opportunities that exist for improvements. For instance, if virtual peer teaching is utilized, one must be selective in the software used as this was a major barrier that students cited. Another opportunity is better timing of activities so students not only have time to learn the material in order to make their presentations, but also have time to learn from faculty-vetted presentations prepared by their peers. Additionally, clear communication regarding the benefits of peer teaching is imperative so that students may better appreciate the purpose of the activity. Finally, the goals of the activity and mode of examination are important when considering how to implement peer teaching in the anatomy lab. For example, if students will be examined on cadaveric dissections, they may place more emphasis on peer teaching occurring in a laboratory setting as opposed to a virtual format. Alternatively, if students are tested using cadaveric images or some other form of instruction that is not laboratory based, then a virtual format to peer teaching may be more favored. Thus, the context of instruction and examination are likely to be important here.

With respect to limitations of the study, one relates to the number of students who participated in the focus group session. Since 6 out of 124 students participated, it is possible that their opinions do not necessarily reflect those of the entire class. Another limitation of the study was the time that faculty devoted to providing timely and actionable feedback to the students. As previously stated, it is important that feedback be easily understood and interpreted by the student ( Henderson *et al.,* 2019). While faculty used a rubric that was adapted to both the video recorded as well as PowerPoint generated teaching materials, some faculty provided more detailed comments while others did not. In addition, students often did not receive this feedback until after the Block was finished. While students could still apply the faculty feedback when generating new materials in a different Block, it may have been more pertinent and useful for students to have applied the faculty feedback to the current Block of anatomy. This would have given student peer groups an opportunity to correct content that was incorrect and/or presented poorly. Furthermore, this format of timely feedback may have addressed a theme brought up by students in the focus group that related to concerns of peer teaching materials being less trustworthy than those provided by the faculty. Finally, a limitation the authors acknowledge is that students were adapting to a new learning environment and modalities of learning in light of Covid-19. This may have resulted in students feeling more uncertain or anxious about their perceptions of learning, leading to students managing their time to ensure that they were using the available resources as best as possible. Considering that faculty conducted virtual anatomical (e.g. donor) review sessions with the students and provided practice questions and labeling exercises with answers and feedback, it is likely that students placed peer teaching materials lowest on their list of anatomical resources.

In conclusion, this study highlights a novel method for introducing peer teaching in the gross anatomy lab. Although certain goals were achieved with respect to better student participation and student preparation of high-quality teaching materials, students did not view this resource as beneficial toward their knowledge of anatomy. It is possible that virtual peer teaching can be successfully implemented if the goals and expectations are clearly communicated, the technology is user-friendly, and the timing of activities as well as the format of feedback is conductive to student and faculty engagement. Although these were not the anticipated results, the authors believed it important to share these findings for anyone considering such an approach in their curriculum.

## Data Availability

Zenodo: Virtual Peer Teaching in the Anatomy Lab. https://doi.org/10.5061/dryad.41ns1rnhc ( Hielscher, 2022a). This project contains the following underlying data: Data file 1: Peer Teaching Grades_Block 1 Back Data file 2: Peer Teaching Grades_Block 2 Upper Limb Data file 3: Peer Teaching Grades_Block 3 Lower Limb Data file 4: Peer Teaching Grades_Block 4 Thorax Data file 5: Peer Teaching Grades_Block 5 Abdomen Data file 6: Peer Teaching Grades_Block 5 Pelvis Data file 7: Peer Teaching Grades_Block 6 Head and Neck 2 Data file 8: Peer Teaching Grades_Block 6 Head and Neck Survey Questions for Peer Teaching: Questions used to guide the focus group interview. Data are available under the terms of the Creative Commons Zero "No rights reserved" data waiver (CC0 1.0 Public domain dedication). Zenodo: Virtual Peer Teaching in the Anatomy Lab. https://doi.org/10.5281/zenodo.6620163 ( Hielscher, 2022b). This project contains the following extended data: Grading rubric for virtual Peer Teaching.docx (to assess Camtasia® (page 1) and PowerPoint ^®^ (page 2) presentations) Course Evaluation Question Associated with Anatomical Resources.docx (Question provided in the course evaluation regarding preferred anatomical resources) Course Comments on Peer Teaching.docx (Student feedback associated with the virtual peer teaching. Color coding in the document represents frequently used terms) Data are available under the terms of the Creative Commons Attribution 4.0 International license (CC-BY 4.0). The raw transcript data from the focus group interview cannot be shared publicly as participants were not informed that their data would be reviewed by anyone other than the group facilitator. To request access, the authors would need a document from the interested party stating their university affiliation and title, correspondence information, purpose for the request, how the data would be used, and how the reader would maintain confidentiality of the participants’ information. We would advise that the reader understand that prior to granting access, the authors would also need consent from all participants in the focus group to share this information. The raw data from the Likert scores related to student preference of anatomy resources (please refer to Table 1) is not available as this data was part of a larger course evaluation which contains names of faculty members which cannot be de-identified.
