# Supplementary material for: Virtual peer teaching in the gross anatomy lab: a format of peer teaching and learning during the COVID-19 pandemic
Source: MedEdPublish (2016). 2023 Mar 9;12:48. Originally published 2022 Jul 6. [Version 2] doi: 10.12688/mep.19178.2 (PMC10126730; doi:10.12688/mep.19178.2)
Supplement: Peer review history

# Peer review: Reviewer response for version 2

**DOI:** 10.21956/mep.21003.r33016

**Published:** 2023-04-24

**Authors:** Valerie O'Loughlin

**Affiliations:** 1 Medical Sciences Program, Indiana University School of Medicine, Bloomington, IN, USA

The authors have made significant improvements to the manuscript - well done!

 There are still a few edits that need to be made in the paper:

1. In the 'thematic analysis of focus group comments', paragraph 1 and the beginning of paragraph 3 are almost identical.  Please edit this section to remove the redundancy.
2. Thank you for including the reflexivity statement. I suggest beginning 'reflexivity was considered in the data analysis' its own paragraph, so this information is not 'lost' in the manuscript.

Other than these two issues, everything else looks great! Nicely done!

Have any limitations of the research been acknowledged?

No

Is the work clearly and accurately presented and does it cite the current literature?

Partly

If applicable, is the statistical analysis and its interpretation appropriate?

Not applicable

Are all the source data underlying the results available to ensure full reproducibility?

Yes

Is the study design appropriate and is the work technically sound?

Partly

Are the conclusions drawn adequately supported by the results?

Yes

Are sufficient details of methods and analysis provided to allow replication by others?

Partly

Reviewer Expertise:

Anatomy education research

I confirm that I have read this submission and believe that I have an appropriate level of expertise to confirm that it is of an acceptable scientific standard.

# Peer review: Reviewer response for version 2

**DOI:** 10.21956/mep.21003.r33017

**Published:** 2023-03-16

**Authors:** Julie Hunt

**Affiliations:** 1 College of Veterinary Medicine, Lincoln Memorial University, Harrogate, TN, USA

I reviewed the authors' version 1 of this manuscript. The authors have made numerous changes which have improved the quality of their paper. In particular, the addition of details on the method of qualitative data analysis and a description of the study limitations are appreciated.

 For transparency, I am on the Advisory Board of MedEd Publish and am a Fellow of AMEE.

Have any limitations of the research been acknowledged?

No

Is the work clearly and accurately presented and does it cite the current literature?

Yes

If applicable, is the statistical analysis and its interpretation appropriate?

Partly

Are all the source data underlying the results available to ensure full reproducibility?

Yes

Is the study design appropriate and is the work technically sound?

Partly

Are the conclusions drawn adequately supported by the results?

Yes

Are sufficient details of methods and analysis provided to allow replication by others?

Yes

Reviewer Expertise:

I have more than 10 years of educational research experience. My own areas of research include the development and validation of clinical skills simulators, the methods of effective health professions instructors, and the reliable and valid assessment of students' skills. I have also published on the application of new technologies in education, including virtual reality applications.

I confirm that I have read this submission and believe that I have an appropriate level of expertise to confirm that it is of an acceptable scientific standard.

# Peer review: Reviewer response for version 1

**DOI:** 10.21956/mep.20545.r32817

**Published:** 2022-12-22

**Authors:** Valerie O'Loughlin

**Affiliations:** 1 Medical Sciences Program, Indiana University School of Medicine, Bloomington, IN, USA

The authors present an interesting study regarding virtual peer teaching.  I have the following suggestions to make this manuscript even better.

1. Introduction and Discussion - The literature about peer teaching could be expanded. There are at least two dissertations from Indiana University that examined peer teaching in the medical gross anatomy lab that should be examined and cited in this paper. (see Dunham and Newman PhD dissertations below) The Dunham dissertation especially has relevance to your study and definitely should be cited. In addition, *Anatomical Sciences Education* has MANY articles about peer teaching in anatomy - why weren't these cited?  (I've put in the search URL for anatomical sciences education and Peer teaching - see citation 3). In addition to the numerous peer teaching anatomy articles in ASE, there is a 2021 article about remote peer teaching that you must cite (see citation 4). Please do a more extensive lit review
2. Introduction - put a citation in the 2nd paragraph when you define reciprocal peer teaching.
3. Introduction, last paragraph - please rework and revise. The last paragraph is more of an abstract summary of the paper - when instead, you should be 'setting the stage' for your research and introducing your research questions. You shouldn't be summarizing the results of your study at the end of the intro. Plus, since you are utilizing qualitative methods, your questions should not have a priori hypotheses.
4. Methods - the single sentence of course details is not sufficient. How many students per donor? What were the assessments in the class? (the type of the assessment can drive the peer learning, and vice versa). What other topics were in the FoCS, and to what extent was anatomy emphasized in this 18 week course? Did it make up 1/2 of the course? 1/4? less? All of this info will have an impact on the instruction and peer teaching of the course. (some of your info about blocks in the peer teaching assignments should be first introduced in the course details section - but in greater detail than what you have).
5. Methods - tools used - in general, you need to provide greater description of the tools you used for the learner. For example, you mention Camtasia but you don't provide specific information about HOW it can be used to create and edit videos, and what format the videos are in. (you should cite the version of the camtasia software used too, by the way).
6. Methods - peer teaching description - it still isn't clear to me about the number of sessions and who did what. When you say that there was 1 peer teaching session in a block, does that mean that EVERY student group prepared a teaching activity for that block - or only SOME of the groups prepared a video for that block, and the others reviewed the videos? Did every group review EVERY presentation, or did they just have to review 1 other group's presentation? Did you 'mix up' which group reviewed which presentation (i.e., group 1 reviewed group 4 in block 1, then they reviewed group 5 in block 2, etc). All of these details are needed for someone to be able to replicate your study.  
7. Faculty grading of student presentations - again, it isn't clear if the peer feedback was incorporated in some way in the faculty grade. Did faculty review the peer feedback when grading a presentation, and incorporate it into their own grade? Why or why not?
8. Thematic analysis description is too brief and incomplete. You need to define thematic analysis for the reader, and explain briefly how it differs from other types of qualitative analysis. You need to describe and explain the six steps of thematic analysis you did - not simply mention you used a six-step thematic analysis. You need to explain to the reader what you mean by 'constructivist approach' (individuals not well versed in qualitative research may not know the difference between constructivist vs. a positivist approach), you need to explain saturation - you need to explain the iterations of your codebook before you reached your final version. (and it isn't clear what you mean by 'coding was done by Microsoft word'. MS word doesn't do qualitative analysis. Clarify.)  Also in the thematic analysis description - you should have a reflexivity statement that discusses the authors' positionality with the research in question, and reflect on how your positionality may affect your qualitative analysis. For more info about reflexivity statements, search in google and you will find lots of good background information.
9. The discussion of your themes is nice! Do remind the reader how many students participated in each group (I had to hunt in the methods to find that info).  
10. Discussion - again, you need to have a more thorough list of citations and better discuss your findings with respect to that expanded list of studies.
  And - you really can't say 'a common concern amongst students' was... if the data you are using is from the 6 students in the focus group. You can say "a common concern amongst the students in the focus group" - but don't conflate your qualitative findings of 6 students to be true for all 124 medical students.  
11. At the end of your discussion, you discuss limitations regarding the *instructional method*, but nowhere do you discuss the limitations of *your study. *You need to have a limitations section. For example - you had only 6 out of 124 medical students participate in the focus group. Can you REALLY assume that this 6 provided you with saturation of your data? You need to mention that it is possible these 6 voices are not representative of the whole class. Were there other types of analyses or data collection you could have done to provide you with more information?  etc.
12. Your conclusion is concise and effective!

Have any limitations of the research been acknowledged?

No

Is the work clearly and accurately presented and does it cite the current literature?

Partly

If applicable, is the statistical analysis and its interpretation appropriate?

Not applicable

Are all the source data underlying the results available to ensure full reproducibility?

Yes

Is the study design appropriate and is the work technically sound?

Partly

Are the conclusions drawn adequately supported by the results?

Yes

Are sufficient details of methods and analysis provided to allow replication by others?

Partly

Reviewer Expertise:

Anatomy education research

I confirm that I have read this submission and believe that I have an appropriate level of expertise to confirm that it is of an acceptable scientific standard, however I have significant reservations, as outlined above.

## References

[1] : Assessing the Peer-Teaching Quality and Strategies of Medical Students within the Human Gross Anatomy Laboratory during Reciprocal Peer-Teaching (Order No. 28547931). *Available from ProQuest Dissertations* .2021; Reference source

# Peer review: Reviewer response for version 1

**DOI:** 10.21956/mep.20545.r32706

**Published:** 2022-10-31

**Authors:** Julie Hunt

**Affiliations:** 1 College of Veterinary Medicine, Lincoln Memorial University, Harrogate, TN, USA

This manuscript describes a peer teaching activity performed virtually during the COVID-19 pandemic in an anatomy course. I applaud the authors for their implementation of this learning activity during a time when instruction was difficult. The manuscript describes negative findings; that is, although student scores on the peer teaching activity were high, students' comments in their course evaluations and focus group suggested that the peer teaching resources were not used by students, and the activity was seen as a waste of time. I appreciate the authors' willingness to report on an activity that was not fully successful, as the reporting of negative findings is helpful in guiding other educators as they consider the creation of potentially similar activities.

 As a member of my college's Institutional Review Board (IRB), which determines the ethical approvals of studies involving human participants, I would have required ethical approval and informed consent for the focus group interviews. I question the authors' statement that this activity falls under operational improvement activities and does not require IRB review.

 Qualitative analysis of the focus group session would have been more reliable if done by two coders rather than one. The methods do not indicate whether the same coder who analyzed the focus group sessions also analyzes the course evaluation comments, or whether those analyzed by someone else.

 Overall, the reliance on student opinions on the value of the peer teaching activity is weak evidence. A stronger study design would have compared the course performance of students randomized to participate in the peer teaching activities to those randomized to non-participation. Alternatively, the authors could have expanded their comparison between learning outcomes and course grades in 2019 (in-person peer teaching) to those in 2020 (with the presently described assignments); however, there were so many other things different between the teaching in 2019 (pre-COVID) and in 2020 (during COVID), that this comparison is likely not valuable.

 The authors did a good job in the discussion of elaborating on the reasons they thought the activity may have fallen short. I would have liked to have seen an overt discussion of limitations and suggestions for future research necessary in this area.

Have any limitations of the research been acknowledged?

No

Is the work clearly and accurately presented and does it cite the current literature?

Yes

If applicable, is the statistical analysis and its interpretation appropriate?

Partly

Are all the source data underlying the results available to ensure full reproducibility?

Yes

Is the study design appropriate and is the work technically sound?

Partly

Are the conclusions drawn adequately supported by the results?

Yes

Are sufficient details of methods and analysis provided to allow replication by others?

Yes

Reviewer Expertise:

I have more than 10 years of educational research experience. My own areas of research include the development and validation of clinical skills simulators, the methods of effective health professions instructors, and the reliable and valid assessment of students' skills. I have also published on the application of new technologies in education, including virtual reality applications.

I confirm that I have read this submission and believe that I have an appropriate level of expertise to confirm that it is of an acceptable scientific standard, however I have significant reservations, as outlined above.
